# Domesticating the condition: Design lessons gained from a marathon on how to cope with barriers imposed by type 1 diabetes

**DOI:** 10.3389/fpsyg.2022.1013877

**Published:** 2022-11-07

**Authors:** Denise Montt-Blanchard, Karen Dubois-Camacho, Stefanella Costa-Cordella, Raimundo Sánchez

**Affiliations:** ^1^School of Design, Pontificia Pontificia Universidad Católica de Chile, Santiago, Chile; ^2^Faculty of Medicine, Institute of Biomedical Sciences, Universidad de Chile, Santiago, Chile; ^3^Faculty of Psychology, Universidad Diego Portales, Santiago, Chile; ^4^Millennium Institute for Depression and Personality Research (MIDAP), Santiago, Chile; ^5^Faculty of Engineering and Sciences, Universidad Adolfo Ibañez, Santiago, Chile

**Keywords:** type 1 diabetes, health psychology, autoethnography, physical activity, coping mechanisms

## Abstract

Through analytical autoethnographic analysis of marathon preparation, this study examines challenges faced by people with Type 1 Diabetes (T1D) who engage in high-performance sports. Autoethnographer and second-person perspectives (T1D runners, family members, and health providers) were collected through introspective activities (autoethnographic diary and in-depth interviews) to understand the T1D runner’s coping experience. Six insights involved in T1D self-management were identified and analyzed with reference to related design tools (prototyping, archetyping and journey mapping). Finally, we conclude with a discussion of how endurance physical activity (PA) such as running helps to “domesticate” T1D, a term coined to reflect the difficulties that T1D presents for PA accomplishment and how T1D runners’ experiences give them an opportunity to overcome PA barriers promoting physical culture and enriching further health psychology studies.

## Introduction

Type 1 Diabetes mellitus (T1D) is a chronic disease affecting children and adults (Diaz-Valencia et al., 2015). T1D incidence is increasing worldwide (Mobasseri et al., 2020). Regular physical activity (PA) in T1D patients improves cardiorespiratory fitness, vascular function, and blood lipid profile (Mobasseri et al., 2020). PA is also essential for blood glucose regulation and contributes to psychological well-being (Edmunds et al., 2007). In people with T1D, PA might involve complex interactions between human and non-human actors (i.e., patients integrating real-time blood glucose monitoring devices) embedded in networked technologies that shape individual experience.

Despite the positive effect of regular PA, only 17.8% of T1D patients are active. Approximately ∼60% of T1D patients do not perform the minimum recommended exercise (Bohn et al., 2015). This lack of PA has been associated with six main barriers to carrying out exercise such as fear of hypoglycemia (Brazeau et al., 2008), insufficient time, deficient access to facilities, absence of motivation, issues around body image, and a general scarcity of knowledge around PA management (Brazeau et al., 2008; Lascar et al., 2014). These impediments to PA accomplishment call for a need to explore new knowledge production sites beyond the medical disability model. From a disability model, individual cure is conceived as the desired future for disability (Krafer, 2013). In contrast, critical engagement, such as the approach proposed by feminist science theory, allows for careful analysis of intimate lived experiences (Forlano, 2017). Thus, critical engagement highlights how T1D impairments are not disabling, even while social environments continue to have an influence on patient care.

Higher exercise intensity leads to a higher risk of hypoglycemia (Adolfsson et al., 2018). This risk is even higher for those engaged in endurance PA, such as athletes – mainly runners – who have an increased trend to lower glucose levels (Graveling and Frier, 2010). In this regard, diabetes-specific barriers to PA, such as hypoglycemia, cannot be addressed by improving patients’ external conditions (as in responses to other patient needs, for example, through improved urban design or addressing visual impairments). Rather, such barriers must be handled by the T1D individual and through self-care management, with the individual solely responsible for any health consequences. Recognizing this, design in health – emerged as a facilitative system to shift toward person-centered care (Pfannstiel et al., 2019, Chap. 3), has been fruitful in developing methods and devices that help users to address their diabetes, especially people engaging in high-intensity PA. Design tools are useful for collecting user-centered research data, synthesizing and analyzing information, communicating results, and designing implications (Martin and Hanington, 2012, p. 6). For example, social identity map, a chart that integrates the position of an individual in the society (Jacobson and Mustafa, 2019), and paper diaries, a document to maintain recordings about events in their life (Weil, 2006), are design tools useful to be self-reflexive around itself and social environment in which PA and T1D are immersed. Journey mapping is also a design tool that helps visualize and illustrate daily activities and can facilitate timely healthcare and promote proactive delivery of patient-centered palliative care. In the T1D context, Riddell has proposed mapping the T1D athletes’ experience (Riddell et al., 2020) that is beneficial to understanding the decision-making process of chronic diseases. As a whole, these design tools bring qualitative data that can facilitate the comprehension of variables affecting T1D training.

In addition, devices for continuous glucose monitoring (CGM) allow an individual to record glycemic variability, something that must be carefully supervised during (and for hours following) PA performance (Tagougui et al., 2019). Thus, data from CGM, expressed by the insulin pump interface as line charts that show the instant glycemic situation, as well as percentage of time in which the blood glucose levels are within the clinical recommended range or Time in Range (TIR, glucose levels 70–180 mg/dl in more than 75% of readings; Battelino et al., 2019), are very useful for user decision-making.

Furthermore, studies in controlled environments have shown the benefit of CGM in avoiding glycemic excursions in people with T1D who participate in sports (Riddell and Milliken, 2011). Evidence from real-life conditions, however, has been studied less and has been restricted to established exercise protocols (Abdulrahman et al., 2018). Such studies are also more complex, even the same PA may have a different impact on glycemic control when performed outside the laboratory (Aronson et al., 2020). There is evidence of how PA can negatively impact glycemic control; however, people with T1D who perform vigorous PA in daily living conditions have been found to achieve adequate glycemic control on training days (Abdulrahman et al., 2018; McCarthy et al., 2020). Thus, it has been proposed that consensus standard targets for CGM be more flexible for training and competition days (Riddell et al., 2020) with TIR recommendations on lower percentages. Hence, athletes should aim for >70% of TIR instead of >75% (Bingham, 2019).

Various design efforts have sought to improve glycemic control, including technological assets and equipment for CGM measurement. However, most people with T1D are still unable to reach a recommended 150 min of moderate-vigorous PA per week (Sallis et al., 2015), calling for a new way to understand T1D people’s needs and practices. In this regard, T1D runners comprise an interesting group for a deeper study to understand how T1D people struggle to accomplish PA goals, perhaps the hypoglycemia risk. Moreover, ethnographic approaches are relevant here since they focus on observing runners as actors who are developing self-care strategies for T1D management.

Combining realistic medicine (Inns and Mountain, 2020), which places the patient at the center of a shared decision-making process, with a user-centered design view can help us understand how T1D people cope with PA barriers, while also recognizing that one-size-fits-all approaches to health and social care are typically not the most effective path for the patient (Chief Medical Officers NHSS, 2017). Human-centered design emerged from the field of industrial design (Bhattacharyya et al., 2019) and has sought to enrich perspectives on self-care in chronic conditions, developing significant patient-centered solutions, alleviating hypoglycemia fear, and boosting confidence between others (Kanstrup et al., 2015; Groeneveld et al., 2018). Such insights from the field of design offer innovative and powerful perspectives to help solve health issues that influence self-care.

Self-care is an ongoing process adapting the self (both mind and body) to new technologies. Integrating information that is wirelessly being generated, sent, and shared between devices, creates a highly specific and unique complex scenario for each patient. In this regard, medical practitioners are called to improve patient care by developing a more personalized approach (Choi et al., 2020). Also, integrated psychosocial care is recommended with the goal to provide collaborative, patient-centered medical care to all people with diabetes to optimize health outcomes and health-related quality of life (Young-Hyman et al., 2016).

Most technological advances developed as tools for better T1D self-care are mainly design artifacts, such as portable blood glucose meters, insulin pens and pumps, enabling improvements in diabetes management. However, technology-based self-care implies creating new interfaces between medical devices and the patient. This creates dependencies and can add to the user’s management burden. The complexity of combining and monitoring quantitative measurements, data transmission, and phenomenological lived experiences generates laborious practices, also known as “data rituals” (Forlano, 2017). Under endurance PA, data rituals can also include nutrition information, insulin sensitivity and kinetics, among others (Colberg et al., 2015). The latter adds further complexity to the challenge of processing all these data.

Even though data displayed from devices are important resources for self-management, devices tend not to match the exercise-specific requirements of PA. For example, closed-loop systems (the automated insulin delivery technology) do not allow users to register what is called “uncovered carbohydrates” that active people with T1D take without insulin bolus to avoid hypoglycemia during PA (Zaharieva et al., 2020). Some users even use interfaces to adapt them to their needs.

Thus, we can see a T1D individual engaging in PA as a “continuous data integrator.” Building a system for optimum patient care, such as software integrating into hardware, requires understanding the strategies that underpin these processes. Crucial here is integrating the role of design, which comprises efficiency, flexibility, development time, problem-solving and connecting diverse functions (Bohemia, 2002). From a diabetes perspective, problem-solving is related to a learned behavior that includes generating a set of potential strategies for problem resolution, selecting the most appropriate strategy, applying and evaluating the effectiveness of the strategy (Mulcahy et al., 2003). Problem-solving is also an essential skill for effective self-management in facilitating diabetes goal-setting (AADE, 2020) and improving individual HbA1C levels (Hill-Briggs and Gemmell, 2007). When individuals succeed in solving their self-identified problems, they gain confidence in their ability to handle future challenging situations (Kolb, 2021). However, approaches to solving complex issues such as minimizing T1D barriers to PA (and building resilience) have not been explored from a research-through-design perspective.

To address this gap, the current study is a human-centered research project, with users as active participants and creators of knowledge (Ku and Lupton, 2020). We aimed to understand the factors that influence the experience of a person with T1D to reach a high-performance sports goal through autoethnographic analysis of the preparation of a marathon as a case study that allows illustrating how to overcome the barriers that the condition of diabetes imposes for the realization of this type of physical activity. We examined the importance of self-reflection and personal experience in developing more effective design strategies. We also applied design tools as research methods to collect and communicate information on personal challenges that a T1D individual has to overcome to complete a marathon. In this research process, design operates as a method to generate knowledge requiring praxis, exploration, and self-reflection (Müller, 2021, pp. 21–29). We conducted an analytic autoethnography of the marathon preparation in order to (i) show PA impact on diabetes management and (ii) to gain a better understanding of intimate personal practices that a T1D person has to go through to achieve a high-performance sport goal, mainly avoiding hypoglycemia. Our findings were analyzed by integrating determinants of health such as emotional (negative mood, stress, and anxiety), medical care (diabetes management and patient empowerment), environmental (quality and friends support, gender influence), and psychosocial factors to evaluate their effect on PA performance and self-care (Anderson, 2006; Young-Hyman et al., 2016). Finally, the study was also guided by constraints – further drawing on design research that highlights how constraints can push abductive thinking (Stickdorn et al., 2018) and boost creativity.

## Methodology

### Participants

A total of 15 subjects over 18 years old were enrolled for this study. Autoethnographer (main study subject) and 5 more runners (second-persons) with at least 5 years from T1D diagnosis were recruited. In addition, 4 T1D subjects with other sports expertise, 3 diabetes health care professionals, and 2 family members of the main study subject were also recruited to complement data triangulation (Table 1). The study considered medium and advanced runners with a training volume of over 21.7 ± 5.3 miles a week and more than 6 ± 3.2 h weekly training, according to categorization from runners’ profiles analyzed by Besomi et al. (2018). Since most of the T1D community are still members (regardless of their age) of Fundación de Diabetes Juvenil de Chile (Chilean Juvenile Diabetes Foundation) and Corporación de Ayuda al Niño y Adolescente con Diabetes (Corporation for Aid to Children and Adolescents with Diabetes), these 2 institutions also assisted with recruitment. This study was developed during 2020–2021 and was approved by the Social Sciences Ethical Committee at Pontificia Universidad Católica de Chile. All participants signed informed consent for interview, data collection and analysis. Due to the exploratory and qualitative nature of this study, the sample selection was emergent (taking sampling decisions according to the analysis of first deep-interviews; Cohen and Crabtree, 2006).

**Table 1:** Characteristics of subjects enrolled in this study.

Subject^#^	Sports	Gender	Age	Years of T1D diagnosis	Profession
1	^*^Runner (Main Subject of Study)	Female	42	39	Designer
2	Runner	Male	31	10	Sales Rep
3	Runner	Male	55	42	Chemical Engineer
4	Runner	Male	48	18	Civil Engineer
5	Runner	Male	42	33	Business Administration
6	Runner	Female	33	10	Business administration
7	Sprinter	Female	23	10	Business Student
8	Recreational	Female	40	33	Public Relations
9	Swimmer	Male	20	5	College student
10	Rugby player	Male	27	26	Civil Engineer
11	Recreational	Female	38	NA	Diabetes Nurse (Health care provider)
12	Recreational	Female	35	NA	Diabetes Psychologist
13	Runner	Male	52	NA	Sports Medical Nutritionist
14	Pilates	Female	67	NA	Autoethnographer Mother (Artist)
15	Athleticism	Female	18	NA	Autoethnographer Daughter (Student)

* Autoethnographer.

# 1–9 are subjects living with diabetes, 10–15 are second persons not living with diabetes.

### Methods

This study uses a human-centered design-focus to understand decision-making in endurance PA in the context of a chronic condition such as T1D.

T1D is a condition that requires numerous, thoughtful decisions and ‘constant self-care behaviors’ (Abdoli et al., 2020) that increase when performing PA. A strategic design management approach helps integrate this complex process, examining design as a new paradigm to arrive at general ideas and methods for enhancing management efficiency (Best, 2006). We focus on identifying those elements in which design can help create sustainable strategic advantages (Borja de Mozota, 2003). For example, by allocating creative assets to communicate the user’s problem-solving strategies, designers may use visualization skills to engender behavior changes (Cooper et al., 2011); useful for a T1D person interested in performing PA. Moreover, introspection is relevant to design as a means for understanding individual experiences (Xue and Desmet, 2019). Thus, we used autoethnography and ethnography methodologies for a better understanding of the complexity of decision-making processes in PA according to experiences in T1D context.

#### Autoethnography

This methodology was selected as a primary method for its interpretive power and its focus on connecting the researcher’s personal experience with cultural and social context as a way to analyze individual practices in more detail (Jones, 2005), helping to understand decisions and self-care behaviors during PA accomplishment.

Thus, to comprehend individual behavior health determinants during PA in T1D context qualitative data was collected through (i) in-deep Interview (interview transcripts that include autoethnographer’s mother and daughter), (ii) Athlete Diary (handwritten notes and figures), (iii) Identity Mapping to understand identity construction (handwritten figures), and (iv) a perception score surveillance. To evaluate individual behavior health determinants (physical activity, glucose control and perceptions) during PA in T1D context we collected quantitative data from autoethnographer. These data included glucose monitoring (time in range) and heart rate (HR zones) parameters. Time in range (TIR) parameters simplifies the assessment of glycemic control and includes the percentage of time with blood glucose values between 70 and 180 mg/dl (ranges clinically recommended); Time Below Range (TBR), which includes the percentage of time with blood glucose values between 54 and 70 mg/dl and those below 54 mg/dl (hypoglycemia); and Time Above Range (TAR), includes the percentage of time with glycemic values greater than 180 mg/dl (hyperglycemia; Battelino et al., 2019).

Regarding HR, it was measured according to five zones relative to the maximum HR of the runner that a smartwatch can measure, and is related to PA intensity (Skinner et al., 1980). This variable refers to how the heart is beating during the exercise, measuring the athlete’s effort. Thus, Zone 1 corresponds to a HR between 50 and 60% of the maximum HR; Zone 2 to 60–70%; Zone 3 to 70–80%; Zone 4 to 80–90%; and Zone 5 to 90–100%. According to their training program, each runner selects the zone that needs to be worked on to avoid overstressing their skeletal and muscular system, an event that happens when the runner is held in Zone 5 too long (Fletcher et al., 2013).

To understand autoethnographic perception of glycemic control (according to PA intensity), a quality Likert perception score (Perception score I) was evaluated in each training phase, from 1 as a negative or very poor perception (not being able to control glycemic variability) to 5 as a positive or excellent perception (being able to control glycemic variability): “Very poor” = 1, “poor” = 2, “fear” = 3, “good” = 4, “excellent” = 5.

#### Ethnography

This method was used to provide further depth to the second-person’s diabetes experiences in PA context, complementing the autoethnographic perspective. We recruited T1D runners, family members, and health caregivers as informants so that we could observe and analyze behavior in the ‘naturally occurring conditions’ that ethnography favors (Belk et al., 1988).

Qualitative data were collected through (i) in-depth interviews, (ii) Design Probe, and (iii) a Perception score Surveillance. In-deep interviews were conducted with 15 individuals. This sample included 6 runners (including the autoethnographer being interviewed by a research assistant, a strategy which was used for instrument validation); 1 swimmer; 1 sprinter; 1 rugby player; 1 recreational runner; and 2 individuals from family environments. To acquire a professional T1D view, we also interviewed 3 health professionals. Detailed questions are included in supplementary material. Design probe was conducted by 1 runner and the probe was designed to explore determinants of health such as emotional and medical care, that are important in diabetes management. This probe included testing a new sport, registering the emotions involved in the experience, and sharing methods for overcoming PA barriers imposed by T1D (done by sharing photos and text messages with the autoethnographer for 2 weeks on a mobile platform). Information gathered as part of this process included insulin intake, carbs load, PA type, time and intensity to control the participant’s blood glucose response and avoid hypoglycemia. A surveillance was designed to evaluate how athletes rated their apprehensiveness about glucose levels during PA (Perception score II). Athletes rated the level of concern about their blood glucose levels during the training steps (*How much does hyper and hypoglycemia concern you in each training phase?*). Qualitative responses were coded as: “*not at all”* = 1; “*a little”* = 2; “*moderately”* = 3; “*quite a lot”* = 4; “*a lot”* = 5. Perception score II were collected from 6 T1D runners and 1 less active subject.

### Procedure

**Figure 1:**
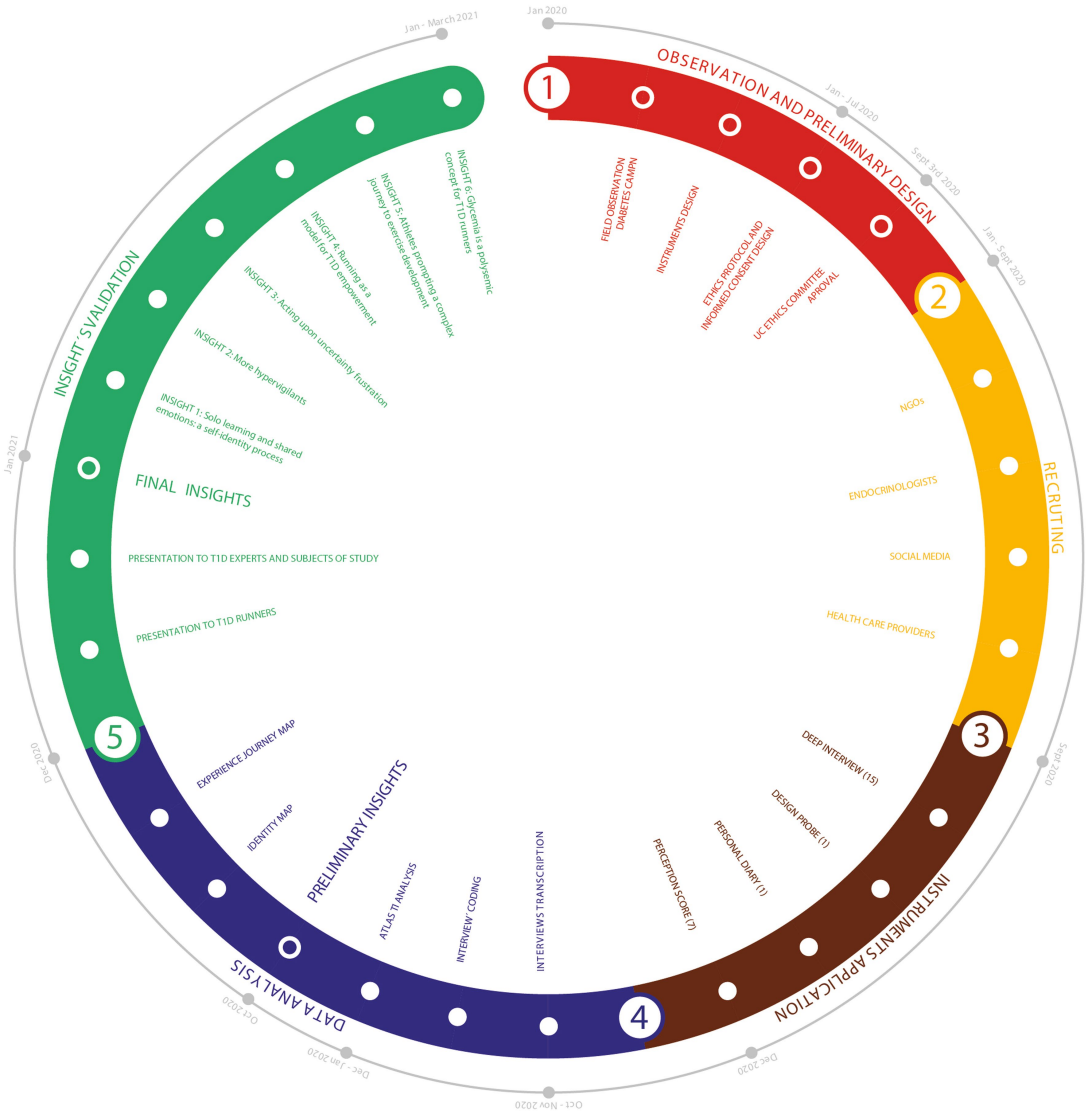
Illustration of the methodological design for recruitment, instruments, data analysis and validation.

This study methodology was divided into 5 phases (see “Methodology,” Figure 1).

In Phase 1: Observation and Preliminary Design; it was the first observation of T1D people in the PA context that would inform posterior study design. Two-day field observation was conducted at a Summer Diabetes Camp organized by Fundación de Diabetes Juvenil de Chile in January 2020. This activity gathered T1D camp participants, team members, and health specialists; the whole group for the week totaled around 90 people. Phase 2: Recruiting; Adult athletes were recruited by social media, and with the support of T1D endocrinologists and health care providers and diabetes NGOs in Chile. Phase 3: Instrument Application; the personal athlete diary was kept throughout the whole study period. These data were recorded primarily in a written daily diary (athlete’s diary) over a month, including insulin regime, food intake, training minutes and glycaemia data from training apps, food photos, and insulin pump screen information on glycemic trends. Comments were also included on each event, the diabetes management strategy used, and changes required for future exercise. Finally, the autoethnographer accomplished an introspection exercise, drawing on information design, to facilitate and systematize the data collection (and to determine subsequent training phases). The Identity Mapping was adapted from the “culture gram” (Chang et al., 2016), which visualizes self-identifiers according to multiple perspectives such as social roles, belonging to groups, and primary culture identities. The information obtained was then color-coded to see how T1D could connect with other self-identifiers. Due to the Covid-19 pandemic, in-deep interviews were conducted online. Also, the design probe was created to avoid potential Covid transmission by physical contact. Our design probe is called “adaptation process for endurance PA.” Since lockdown rules did not allow for outdoor training, a second-person participant used a “stationary bike” which they had not used before. At the end of design probe data collection, autoethnographer and second-person discussed their experiences collaboratively using “visual research” (Bestley and Noble, 2016) – as an instrument for articulating processes, which involves the analysis of icons collected for a deep understanding of PA challenges and barriers. Phase 4: Data Analysis; Deep interviews (autoethnography and ethnography) were analyzed by ATLAS.ti Version 8.4.3 (1077), gathering the basis for preliminary insights, which were further analyzed with triangulation. In this study, triangulation reflects an attempt to secure an in-depth understanding of the phenomenon in question. Triangulation was addressed from the following perspectives proposed by Flick (1992), “data-triangulation” or the combination of different data sources that are examined at different times, places and persons (perception score surveillance, second-person *deep* interviews and feedbacks; Carter et al., 2014), presenting a crosstalk of points of views; “investigator triangulation,” which means the employment of different observers or interviewers to control or correct the subjective bias from the individual and “methodological triangulation” or “technique triangulation (autoethnographic diary, identity map, design probe). In addition, design tools were used to communicate preliminary insights through charts, an identity map, and a journey map. Phase 5: Final insights; the insights were validated in dialog with the second-person research participants and T1D health care professionals (Figure 1).

### Statistical analysis

In data triangulation, the statistical analysis was defined by the nature of the quantitative information obtained both from the autoethnographer and second person’s data (Denzin and Lincoln, 2007). Since the quantitative data obtained presented numeric variables normally distributed, with perception scores being ordinal and with no outliers found, we used *Spearman* correlation. This test was applied to evaluate if the perception of glycemic control (Perception score I) measured during training (training phase 2) correlates with HR zones, and when measure post-training (training phase 5) it was analyzed with glucose control parameters (TIR, TAR, and TBR). *p* value < 0.05 was considered significant. Perception score II and Training phases were graphically analyzed in seven athletes.

## Results

As previously reported, glycaemia during exercise can vary inter-and intra-individually, and It depends on various factors such as exercise modality and intensity, nutritional status, time of insulin injection, or pre-exercise glycaemia level (Tonoli et al., 2012). This background complicates the scenario for T1D athletes to reach their sport goals, generating various elements to consider for decision-making before, during and after a competition or training that also involves different users’ perceptions. The following insights illustrate design lessons gained from a marathon.

### Solo learning and shared emotions: A self-identity process

Exercise or any deliberate practice gives the ability to choose what risk to take. In particular, running puts individuals in considerable deliberate difficulty or “deliberate practice” of working on something that is not easy. In fact, obstacles such as any challenging task have shown to be more productive in problem-solving than hours of repetition of familiar actions (Ericsson and Harwell, 2019). However, when we add T1D into the equation, running could be considered a risk; yet despite exposure to a significant chance of adversity, it favors resilience development (Hilliard et al., 2017).

In this study, T1D people tend to be motivated to start intensive training by contextual situations associated with childhood experiences that can fuel the will to run, e.g.:

*“I used to be good at running when I was in school, but I still remember that gym teacher telling me to slow down, to do less just because of my diabetes” (T1D Runner, 42 years old).*

Further, powerful negative emotions such as a severe car accident because of hypoglycemia *(T1D Rugby Player, 27 years old)*, or losing a T1D friend (*Autoethnographer*) prompted them to pursue endurance exercise. Similarly, positive emotions or memories can trigger intensive PA, such as a relative who instilled courage *(T1D Runner, 26 years old)*. Thus, “diabetes resilience,” defined as good psychosocial and health outcomes (Hilliard et al., 2017), is not a personality trait given with the condition; rather, it tends to be built out of difficulty or risk.

These experiences help us to recognize that resilience is a multidimensional topic that involves the patient support system and strengths. As the Psychologist interviewed stated, “resilience is built considering very important psychosocial factors including education, family support and socioeconomic status; they all help to overcome difficulties and educate the patient on how to stand up after failure.” Therefore, running can contribute to developing resilience, learning how to make decisions, and tolerating frustration, all of which could be considered resources to overcome T1D PA barriers and risks. Running can also help individuals to understand that no disease-based labels such as “diabetic” should restrict their possible activities.

Furthermore, a gap currently exists between the promise and the reality of diabetes care. Practical interventions to facilitate collaborative relationships and foster patient-centered practices are key to closing this gap (Funnell and Anderson, 2004). As intensive running demands the user to be constantly self-aware of health signs and to process them for decision making, a lot of time is dedicated to self-reflection. This introspection supports forms of self-discovery that help with identity development (Waterman, 2011) and which function as a resource to cope with T1D (Luyckx et al., 2008). The studied experiences showed diabetes lived from past perspectives, impacted positively on self-care activities. This was confirmed by an athlete interviewed, echoing the idea of refusing to let diabetes interfere with his sport performance. He was able to lift the highest weight of all his team, even though he had the lowest height. He not only wanted to be better, he wanted to be the best, regardless of diabetes (*Rugby player, 27 years old*).

This was also confirmed by the autoethnographer’s family members, one of who said: “When you run you are not competing against others, you are on a race to beat diabetes.”

**Figure 2:**
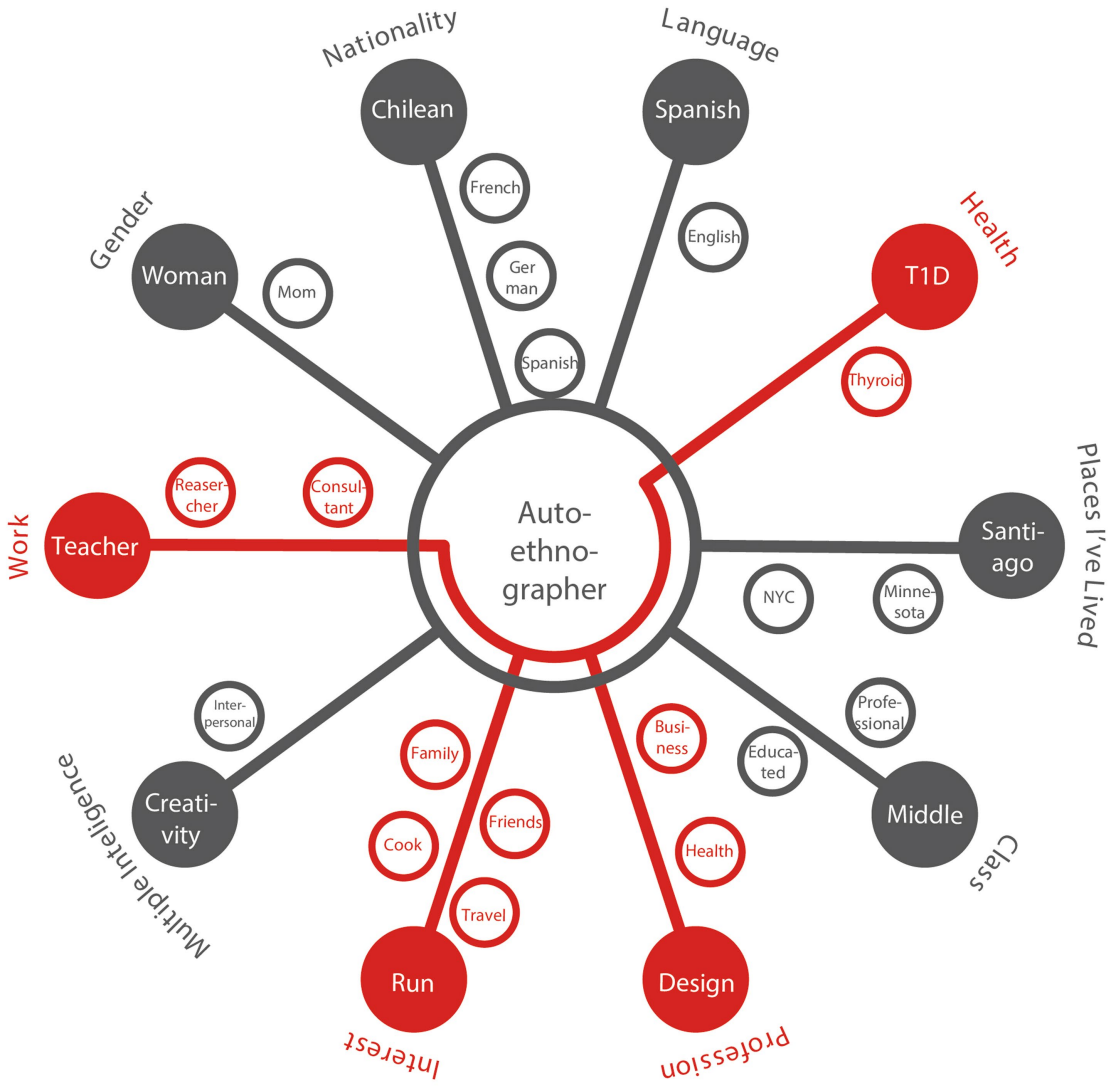
Identity map visualizing self-identifiers and social perspectives.

To delve deeper into the idea of T1D becoming an influential part of the runner’s identity, the autoethnographic identity map adapted from the identity culture gram developed by Chang et al. (2016) shows how PA challenges become a self-identifier for those with T1D (Figure 2).

“*PA hours are a powerful time for my identity, because you only run 1 h a day, but that hour accounts for one of the activities I love the most, and it allows me to be part of an amazing group. Running also gives me a sense of purpose*” (*Autoethnographer*).

Diabetes influences and extends the hours dedicated to running and also can determine whether the sport can be developed or not. With the idea of not letting the condition undermine the athletic passion, T1D starts influencing an essential realm of identity:

*“Diabetes started blending into my identity through running, it also moved into my work and is making me utilize my creativity as a primary self-identifier”* (*Autoethnographer)*.

These findings put running (interests), creativity (multiple intelligence) and design teacher/design researcher (work) as self-identifiers (Figure 2), fixing with the concept established by Chang et al., that relates a self-identifier with knowledge, skills, competence or emotional attachment to function as a member of a group (Chang et al., 2016).

Considering T1D runners as part of a group, most of them claimed to pass through PA in the diabetes context as a solo experience because they have not had opportunities to discuss the topic of diabetes and sports. Runners sense PA as a solitary trip, even though health determinants, such as experiences and emotions are similar among these individuals, particularly those that integrate T1D self-care into a PA lifestyle:

*“I have been very lonely; I have read a lot, but to share with someone who says these are the guidelines and look at this? No, I have developed it by myself*” (*T1D Runner, 42 years old*).

Another participant talked about a very dense spreadsheet where he registered every data that had an impact on his diabetes and helped him with his self-care (*Rugby player, 27 years old*).

Runners stated that they need additional time to include diabetes care (such as analyzing and making insulin and nutritional adjustments) into the general training routine. Hence, there are many self-observation and introspection hours that help identify the patterns and to take actions that improve diabetes self-care (so that T1D does not become a constraint on exercise).

“*It is like the individual extra cost you have to pay to earn the runner identity*.” (*Autoethnographer*).

These declarations suggest that running improves illness integration into identity construction around diabetes acceptance.

### Hypervigilance

Recommendations for psychosocial care of T1D patients involve monitoring the patient’s performance of self-management behaviors and psychosocial factors impacting the person’s self-management (Young-Hyman et al., 2016). When intensive PA is added, the process is more demanding, requiring frequent testing, insulin adjustment, and tighter control of glycemia that can build a sense of hypervigilance against hypoglycemia (Trief et al., 2013). With currently available technology such as insulin pumps and integrated glucose monitoring, a new set of information is available for patient use, integrated into daily living conditions as data rituals (Forlano, 2017).

With T1D, endurance training experience has to be thoroughly planned. In this study we observed that runners tend to monitor themselves 24 h a day, 7 days a week; a behavior consistent with a hypervigilant profile. In fact, some runners mention their physician suggesting “relaxing” and not watching the pump screen too frequently:

*“What my diabetologist told me was not to look at the pump all day. what matters is the complete movie, if some points are off track, as long as they are isolated and not recurring .. no harm will be generated!” (T1D Runner, 33 years old).*

This experience resonates with other patient declarations. *“Even with a different diabetes specialist, the advice was just the same, try not to be watching the pump screen so often” (Autoethnographer).*

Hypervigilance is a behavior that involves an enhanced or exaggerated search of environmental stimuli or scans for threatening information (Rollman, 2009). It is associated with elevated or clinical anxiety levels. Within this context, glucose monitoring and data rituals can be both useful and overwhelming at the same time. Indeed, glycemia values on certain ranges not only activate insulin pump alerts, but also threaten T1D runners with a sense of fear or anger in response to data:

*“As a runner, you certainly do not want to be interrupted while training, so whenever you check your pump, what a great feeling you get when your glycemic trend is steady”* (Autoethographer).

Particular attention should be paid to this topic. Adults with diabetes have a 20% increased prevalence of anxiety disorders than those without diabetes (Smith et al., 2013) when compared with the highest generalized anxiety disorder rates. Women, youths, people with longer diabetes duration, and those with additional medical conditions are at the highest risk (Groot et al., 2016).

### Acting upon uncertainty and frustration

It can be extremely challenging for individuals to balance all the factors involved to improve glycemic control around exercise (Colberg et al., 2015). It can be even more challenging when confronted with a new PA. T1D runners interviewed stated that the idea of doing something with high metabolic demands for the first time could be scary, since there are too many uncertainties on how to avoid the risks involved. Usually, T1D people develop their strategy during PA to avoid hypo and hyperglycemia through trial-and-error and several individual attempts (Finn, 2018). However, as Perkins & Riddell state (Perkins and Riddell, 2006), running is a complicated process requiring a lot of motivation and effort from the individual and their family members; the latter may also sense the runner’s difficulties, e.g.:

“*You know what I think is what frustrates you most about running with T1D? It’s when you do not know why, when you cannot tell why things happen*” (T1D Runner’s daughter).

This uncertainty feeling was also confirmed by another subject who stated that she was eager to learn whether what happened to her during running was, or was not, related to diabetes. For example, when feeling more tired:

“Is this diabetes-related, or is it normal athletic exhaustion?” (Runner, 33 years old).

Besides, runners’ uncertainty is nurtured by the fear caused by the unknown, such as hypoglycemia fear – thus becoming a barrier to exercise. In a study of newly diagnosed patients, hypoglycemia was feared even though some participants had never been physically active (Kennedy et al., 2018). In this study, a diabetes psychologist interviewed confirmed this background:

*“I believe that very few [patients] have experienced severe hypoglycemia and most people are afraid of it. In addition, since patients debut with T1D, people from both their family and health care professionals speak to them demonizing hypoglycemia” (Professional 2).*

High uncertainty is a stimulus for seeking information in anticipation of future interaction (Berger and Calabrese, 1975), which could also be viewed from a design perspective: “*Design is always directed towards an (uncertain) future and helps shape it”* (Simon, 1996, p. 111).

In this regard, self-experience in the T1D running context offers an excellent opportunity to apply and test recommendations given by physicians. Hence, T1D runners try to control the uncertainty by systematizing their training routine, developing methodical records, or keeping track of their blood glucose data during training sessions. As one participant put it:

“At first, I used to write everything down, but once I got the system working, I no longer needed to keep registering information” (Runner, 50 years old).

**Figure 3:**
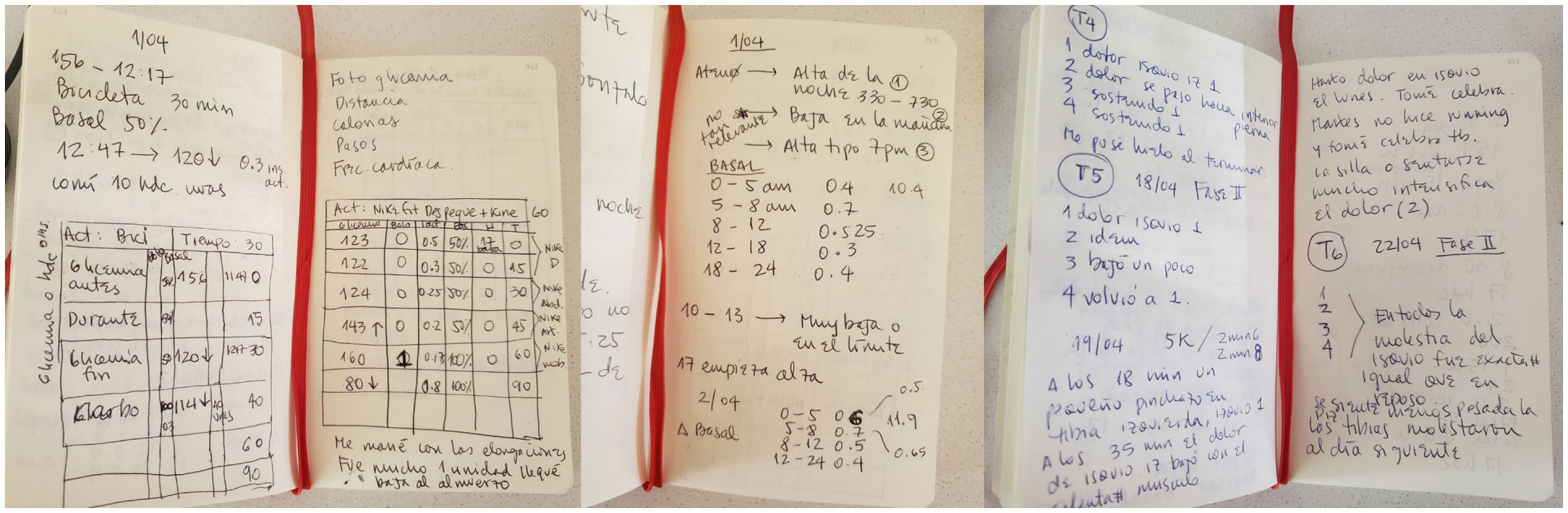
Autoethnographic diary pages showing representative notes of PA management.

Moreover, most of these records are not necessarily shared with each patient’s endocrinologist. However, they provide informed decision-making knowledge (see Supplementary Table 1). The way in which data is displayed in the autoethnographer diary (Figure 3) shows how it can become an information design artifact that enables finding causes for the uncertainties that frustrate patients, helping T1D athletes become their own efficient caregivers.

### Running as a model for T1D empowerment

Empowerment is a psychological state that occurs as a result of effective communication (in health care), and which acts as a determinant of consequent participation, self-management and self-care (Small et al., 2013). Furthermore, empowerment in long-term conditions has been associated with expert-patient initiatives to improve diabetes management (Wilson, 2005). In this study, T1D runners were also observed to increase their empowerment through PA accomplishment, leading to self-care practices and disease coping. These findings show that psychosocial aspects of T1D are an essential field for further study in future research.

On the contrary, emotional exhaustion and frustration with diabetes self-care behaviors refer to a negative psychological state known as “burnout” (Stewart et al., 2019). Burnout is related to diabetes distress (Abdoli et al., 2020) and individuals losing their sense of empowerment. As Abdoli et al. (2020, p. 6) states, some patients view themselves as a “slave” to blood glucose numbers because those “numbers and algorithms” were the “guiding principle” of their lives and the “key indicator of perfect diabetes management.” In this study, some runners feel the constant pressure of glucose monitoring, especially during their training. When they cannot check their glucose levels during a race (for example, due to a failure of diabetes devices), they feel a relieving sensation, improving their focus on the running routine:

“*On kilometer 3 (1.86 miles) of the Chicago marathon, I lost Bluetooth signal, which means my insulin pump could not read the continuous glucose monitor sensor transmission, giving me no chance to check my blood sugar during the rest of the race. It had happened to me before, on crowded races, there is some level of signal interference. So, I had prepared myself for it by spot hypoglycemia just checking on my body sensations and having a running protocol well thought-out and tested on 30 k (18,6 miles) runs twice before. So not being able to get any info from my pump, it had no purpose for me to be constantly checking it and for the first time my attention was set entirely on enjoying the run with an overwhelming sense of control on T1D*” (Autoethnographer).

T1D people with fewer perceived barriers to PA have greater T1D auto control (Brazeau et al., 2008) related to diabetes care. Thus, people with T1D who are able to manage PA barriers are more able to cope with other problems (Brazeau et al., 2008). In this study, runners felt they must perform several self-care conducts successfully to practice sport, which represents a major challenge for the athlete. For example, the challenge of maintaining glucose levels within range, in addition to their running goals. Although this could lead to athlete burnout, getting optimal glucose levels during workout and performance promotes beneficial disease-coping, building diabetes resilience. Running puts the T1D runner in considerable deliberate difficulty or “deliberate practice” of working on something that is not easy, impacting other aspects of athletes’ social lives. For example, runners mentioned that if they can deal with a marathon’s demands, they also will handle diabetes issues:

*“When you reach a certain sport goal, such as finishing a marathon, it feels like any diabetes problem is way smaller than all you had to go through to reach the point where you are”* (Runner, 31 years old).

Hence, running raises T1D empowerment, promoting self-care with self-confidence for improving their health.

### Athletes’ complex journey to exercise development

**Figure 4:**
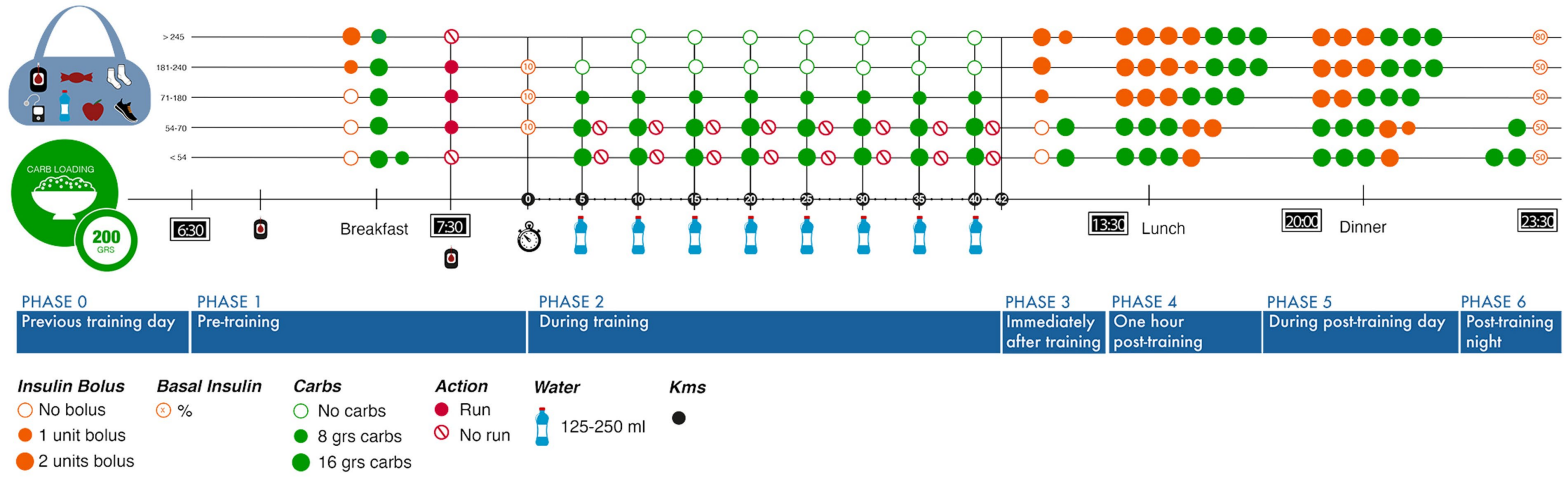
Journey map running designed according to training phases and glycemic control requirements.

T1D is a medical condition involving rigorous decision-making with constant self-care behavior (Abdoli et al., 2020). Analyzing different variables affecting exercise decision-making, this study focused on the relevant elements that define the user experience to avoid hypoglycemia using the autoethnographer’s previous marathon experience. Then, a journey map was designed to visualize the runner’s diabetes management strategies. This was also helpful, to showcase different possible scenarios according to glycemic levels, carbohydrates, insulin bolus and meals, ranging from the previous workout day until the post-workout, reaching the following 24 h (Figure 4). This journey map shows the network representing the challenge for athletes. Further, glucose monitoring was crucial to structure this map, highlighting glycemic levels (>240 mg/dl) or hypoglycemia (<54 mg/dl) at pre-workout (stage 1) that might influence non-running decisions:

*“When I go running with my T1D friend and she hits a very low glycemia, I am the one who does not want to keep up and wait until her levels are up and safe again. So, it is personal. However, she feels relieved to have a low while running with me”* (Autoethnographer).

The idea of getting closer to a fixed running model for a suitable T1D self-care is tempting; however, this is not possible following realistic medical perspectives. Nonetheless, the experience map should provide an overview of what such a process entails and how to modify it:

*“I get the idea that the next one (marathon) it’s going to be easier, still challenging, but you already have a system”* (T1D Runner’s daughter, 18 years).

To adapt this model of journey map for training conditions, the autoethnographer replicated training phases analysis. Due to COVID-19, the autoethnographer changed her training routine to follow confinement restrictions (and also due to a tibial stress injury), utilizing a treadmill with shorter running periods to recover. In order to have a better understanding of training phases, a perception score (Perception score I) was assessed according to PA intensity (heart rate zone). It was observed that low-intensity running was less challenging than high-intensity running in terms of glycemic control – even though all cautions were taken with carbs loading and insulin regimen. Also, an inverse correlation was found between staying in extreme PA intensity (Zone 5) and a lower perception score of glycemic control (Supplementary Figure 1).

“*When I saw the results, I was getting for high-intensity PA, I realized my carbs would need to be adjusted and it was time to call my nutritionist for advice*” (Autoethnographer).

Following the training phases model, achieving better glycemic control was more efficient. Thus, integrating qualitative and quantitative data is useful to improve the maintenance of glucose levels during exercise in T1D runners:

*“My heart rate frequency and glycemic control had never been studied before, and it certainly helped me communicate my concerns to my healthcare provider about how crazy my blood sugar was going using the same insulin and carbs strategy I had used before. As he is also a runner, he easily discovered what I was doing wrong. None of these would have been possible without integrating qualitative and quantitative data”* (*Autoethnographer*).

Then, when the autoethnographer shared the journey map model with second-person participants to validate it, the subjects were able to realize the whole picture and elements that compound their training:

“*It is amazing to visualize our process and how many decisions we need to make for something that might be so simple as running. I am impressed, and by the way, I think this makes us really cool”* (T1D Runner, 33 years old).

Therefore, data visualization of the variables involved in the T1D running experience as an integrated unit reduces uncertainty, guides the search for information, and resonates with other users—thus facilitating learning for diabetes self-care.

### Glycemia is a polysemic concept for T1D runners

Polysemy is a linguistic concept related to the several possible meanings of a word. In this context, polysemy was used to understand how glycaemia levels can have different meanings according to PA training phases or personality characteristics in T1D.

Some personality traits were prominent among the T1D runners, according to the in-depth interviews applied in this study (perfectionist, methodical, resilient, self-demanding, extremist, hypervigilant, competitive, hyperactive). Some of these traits were not different from any other athlete, such as competitiveness and self-demand. However, hypervigilance, fear and perfectionism are all specifically related to T1D. Some athletes reported to their health provider that hypoglycemia fear makes them stop their training routine to avoid a low blood glucose episode:

*“What the athlete wants is to have glycemia as normal as possible, to have optimal metabolic control, such as if they wanted to see themselves as non-diabetic persons. Being lower, you are going to be closer to normal than being high. I think it has to do with their personality. They want to be perfect, even though that is very difficult”* (Nurse).

**Figure 5:**
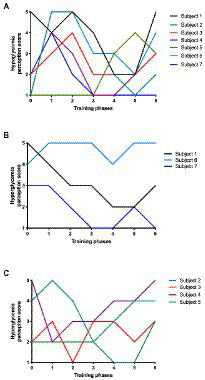
Hypo and Hyper glycemia perception score during training phases. **(A)** Hypoglycemia perception score according to training phases for all enrolled subjects (*n* = 7); **(B)** Hyperglycemia perception score for women (*n* = 3); and **(C)** males (*n* = 4) according to training phases.

A contradiction was found among T1D runners. Their deepest fear is hypoglycemia, being associated with anxiety and mood (Seaquist et al., 2013); yet, when the total perception score of hypoglycemia was studied, hypoglycemia had a lower score (2.5) than hyperglycemia (3.2), suggesting that T1D runners preferred low glycemic levels than to tolerate a hyperglycemic status (Figure 5A), despite the clinical risk.

*“In my case, it scares me more, but I have to admit that I have seen myself and if you have to make me choose, I prefer a low one to a high one… on a high, it takes two hours to get you back down, and running with a high is uncomfortable. One feels the body much heavier”* (Autoethnographer).

There is an objective clinical view of glycemic levels, but the perception of these levels changes according to the subject context. In this sense, both hypoglycemia and hyperglycemia can be polysemic, implying that glycemic data can have a different meaning according to the athlete’s training phase, impacting mood and anxiety. Athletes might therefore frame different emotions evoked around blood glucose levels, but such knowledge remains vague. To address this, we studied autoethnographer perception (*Perception score I*) associated with glycemic range during exercise stages. Perception score correlated inversely with the time below range (Supplementary Figure 2). This finding suggests that positive perception is related to minor hypoglycemic episodes, which confirms the idea of fear around hypoglycemia.

It is also interesting to notice how diverse perceptions (*Perception score II*) were found for severe hypoglycemia (50 mg/dl) according to the training stage. In pre-training and training phases (Phases 0–2), the mean average score tended to be higher (2.9) than post-training phases (Phases 3–6; 2.2; see Supplementary Table 2). Hence, if hypoglycemia happens in pre-training or during training phases, the running could be compromised being much worse than after training phases.

The perception of hyperglycemia was then studied according to gender, finding that women tended to be more worried about their hyperglycemic events (Figure 5B) than men (Figure 5C). Also, hyperglycemia fear emerged as a main PA barrier element that was evident for healthcare providers:

*“Women are much more rigorous and much more methodical in the matter of self-control, exercise, sport and being in shape. Most of the patients I recommend for psychological support are women”* (Psychologist).

Thus, athletes’ perception of hyperglycemia’s interference might depend on gender, consistent with the anxiety prevalence on T1D, where women are at the highest risk (Smith et al., 2013). This different gender-dependent perception reinforces the glycemic levels as a polysemic concept, which changes according to the context and intrinsic characteristics of each subject.

Furthermore, each insight generated during this study can be identified in the introspective first-person experience:

*I woke up around 5 am that morning and had put alarms during the night every 2 h to make sure my glycemia was completely under control. I needed to make sure no insulin was on board for the marathon. After almost 2 years of training to get there, I certainly did not want to mess things up when they were under my control. You need to be at the marathon almost 2 h before the start to get your credentials, put your stuff on lockers, and then wait for at least an hour on your assigned spot. I had my breakfast snack with me, a plain yogurt with 10 grams of carbs, and I ate it 20 min before the start, my pump was showing a nice 109 mg/dl glycemia. I was full of anxiety. What was about to start was something I had never done before, and my dead friend’s memory gave me all the strength I needed.*

*As we were given the go, I watched my pump and it was showing 108 mg/dl (in range, but on the lower end), so I took a “precaution” chew and started running (Glycemia is a polysemic concept for T1D runners). The first 3 km were just wonderful, the weather was perfect and the city’s cheering and its energy, the runners and the thousands of spectators were overwhelming. Before I got to kilometer 4, my insulin pump stopped showing my glycemia. The bluetooth saturation was so high because of all the sports watches that I could not get the sensor signal into my pump. Fortunately, I had trained for this since it had happened to me on the same races before, so I had practiced blind running to get to feel my glycemia while running. So, I did not panic at all, my protocol was to get 8 grams of carbs every 5 k and I totally trusted the hard work, especially by my marathon runner nutritionist, that has put into getting me to where I was (Solo ride shared emotions). So just like that, my anxiety for data was gone (Hypervigilant). I had to trust in my body and follow the protocol. For the first time ever, my mind was only concerned about enjoying the run, no alarms, beeps, or demands from the pump, my diabetes was completely silent, giving me no burden at all.*

*On km32, my quads felt different, heavier, a feeling of dizziness came in and I could figure out what that could mean, it felt kind of hypoglycemia, but I wasn’t quite sure. Just in case, I took an extra chew, 8 grams of carbs, and kept going (Acting upon uncertainty). Only after the marathon I learned about hitting the wall, which refers to depleting the stored glycogen and the feelings of fatigue and negativity.*

*I crossed the finish line at 4:00 h, my exact goal. Exhausted, I immediately asked for a medical assistant to check my glycemia, the reading was 232 mg/dl. Goal accomplished both as an athlete and as a person with T1D (Glycemia as a polysemic concept), I felt enormously proud, this wasn’t competing with me or with others, this was a way to show I was in control of my condition like I had finally domesticated Type 1 Diabetes (Running as a model for T1D empowerment)*.

## Discussion

This study shows how running boosts resilience in T1D people, findings that are consistent with other investigations, as stated by (Hilliard et al., 2012), in which traumas and challenges mobilized and motivated patients, impacting positively on T1D management and self-care. To understand running performance under T1D condition, a proven beneficial design tool for patients and health care was utilized to create a journey map specific for runners. Previous road maps have been reported for Type 2 diabetes and T1D people mainly to assist clinicians in reaching glycemic control goals of their patients (Jellinger et al., 2007) and for self-management education (Der Molen et al., 2017). In this study, the journey map proposed is applied to a PA context, showing phases and variables that need to be considered and controlled by an athlete with T1D. These are aligned with what Riddell et al. (2020) showcased for a training or competition day. In addition, shared emotions between T1D runners (with respect to journey map visualization) suggest that the journey map tool could be useful for self-management not only for the running community interviewed for this study but also for the T1D community worldwide.

Since it can integrate information in a coherent manner, the journey map could also enable patients to become active learners who seek out information and are assertive in their pursuit of understanding and new information (Kolb, 2021). As stated by Fisher et al. (2005), behavior changes and improved problem-solving skills can be facilitated by learning from previous choices and then revising plans based on information gained. From the health care team perspective, the journey map could be aligned with the model for self-care behavior presented by Kolb (2021), because it helps track more variables, sets which information to track, evaluates and suggests the tracking frequency, in order to prevent data overload and decrease time burdens associated with interpretation.

Considering the journey map as a design tool to address a marathon training experience, it could help to “domesticate” T1D, a term that is proposed to understand how this condition can be handled. Domestication is a complex concept related to human social evolution that concerns communicational, cognitive and emotional plasticity, improving behavior, living in community and detriment of aggressiveness (Shilton et al., 2020). In this line, diabetes can also be considered aggressive (for example: during hypoglycemia and hyperglycemia), when it starts screaming, through beeps and sounds from CGM devices, or what Forlano (2017) describes as distressing moments. Continuing along these parallel lines of thought, experimental results suggest that *tameness* (Agnvall et al., 2015) and a *less aggressive* behavior and appearance drive animal domestication (Kaiser et al., 2015), improving their approach to humans.

In this sense, T1D could be compared with an animal that must be domesticated, and within this process, exercise may be a helpful resource as a routine activity that gives more chances to adapt strategies to tame aggressive diabetes behaviors. In this study, when subjects are involved in running, they show what could be considered as domestication practice over their condition when they describe a feeling of empowerment *over* T1D. This possibly relieves the burden of operating in chronic diseases, that has been related with *a slower speed of movement about barriers over which one has little to no control and might need more time to accomplish something or to arrive somewhere”* (Krafer, 2013, p. 45). Hence, T1D people training for a marathon can learn to anticipate diabetes outcomes and patterns because it gives plenty of exposure by trial and error, and enhances problem-solving processes – which involve problem identifying, alternative solutions development, solution selecting, implementation and evaluation (AADE, 2020). These phases can be facilitated by the journey map proposed in this study as a tool to help the domestication process of T1D.

Further, domestication is a process that involves the formation of a mutualistic relationship between human beings and the environment (Price, 1999) and is crucial to boosting adaptation. It is possible to expand this notion to diabetes through the identity map construction presented in this paper, where running promotes the merging of T1D with other identity dimensions, similar to what Chang et al. (2016) show through the culture gram to interconnect culture and identity. In the same manner, when running and T1D are considered as more than just single identity trajectories, they can be viewed [as Wenger (1998) put it] as a nexus of multi-membership that could connect condition and identity. Similarly, the identity map contributes to illness acceptance, as a concept related to the lack of negative reactions and emotions connected with the disease (Cybulski et al., 2017). In this line, low diabetes acceptance is more related to insufficient diabetes self-care and self-management, with poor glycemic control (Schmitt et al., 2014; Oris et al., 2016). The marathon experience and training involved served to integrate diabetes as a beneficial component of personal identity, an internal process of self-knowledge that is facilitated by running. This fits into the design concept of camouflage, aimed to blur and blend, shifting the focus from illness to identity (Kanstrup, 2014).

Moreover, pursuing a goal such as a marathon can be viewed as unattainable, challenging and absolutely out of reach for most diabetes’ patients. There is, in fact, plenty of literature to explain those barriers (Brazeau et al., 2008; Lascar et al., 2014; Finn, 2018; Kennedy et al., 2018). To improve this outlook, the marathon experience from a design perspective allows us to disaggregate the process and helps to identify lessons or recommendations that can be easier or more feasible to apply to a larger T1D audience. Thus, the T1D marathon runner may act as a good example to motivate other people with diabetes to engage in PA.

## Conclusion

This paper presents six design insights to understand and raise valuable information for patient self-management around glycemic control in T1D runners, with the potential to be shared with stakeholders to improve their communication, collaborate, and co-create better user-centered diabetes management strategies.

Moreover, self-care and sports goals facilitate and allow for internal and external validation in T1D context: internally because the training process helps to get a deeper understanding of the physical and emotional self; and externally because sports competitions are also social events that can be shared and celebrated with others. Thus, endurance PA can be taken as an empowerment tool to raise self-care efficiently with T1D.

Furthermore, the term “diabetes domestication” is proposed as a positive adaptation process pursued by T1D people and prompted by running. Moreover, glycemic control is confirmed as the main challenge for T1D patients, and it is a key indicator of achievement in the domestication process.

Future studies involving similar design methods with T1D subjects engaged in endurance PA could further examine ‘domestication’ by testing models for glycemic archetyping, hypo and hyperglycemia, mapping the journey of other sports and evaluating gender differences that were identified in this study.

## Design recommendations for diabetes care

Design recommendations highlight the applicability of using design tools to find out more about the changing context within which T1D runners are immersed. Such recommendations are intended to help ensure that outcomes are future-proofed (Chief Medical Officers NHSS, 2017), allowing the patient to take a step back and commit more time to discovery. This is critical for the initial stages of problem-solving and consequently for finding better solutions for diabetes management. In this section, *design recommendations aligned with empowerment-based strategies for diabetes self-management* are proposed for self-care considering each of the six insights listed above. These recommendations are based on the experience in Chile; however, they may also be of use in other countries for runners with chronic illnesses who need real-life experiences and suggestions that they can relate to. They will also be of interest to design researchers and health providers interested in a better connection with patient needs and in developing new strategies for self-management. This is of particular contemporary relevance since the number of endurance runners with T1D has been growing in recent years, according to the global grassroots movement “Type One Run” (Beyond Type One, 2021).

### Sharing and learning

In contrast to medical perspectives focused on the individual self-management of health data, the concept of sharing is collaborative and qualitative (Kanstrup et al., 2015). In order to share, the runner first has to understand their own identity as a participant of a group, and the identity map proposed in this study gives the runner the opportunity to define how their character is merged with the T1D and to guide self-introspection, giving them a base to share. Consequently, in this study, instruments used for data collection and data visualization facilitated sharing of both qualitative and quantitative information between T1D runners and health professionals in a collaborative manner (see Insight 1: *Solo learning and shared emotions insight*). This insight is reinforced by the design concept proposed by Kanstrup et al. (2015) of sharing chronic illness, which foregrounds users’ active participation, mutual engagement, joint enterprise, and a shared repertoire as a basis for negotiating meaning in practice among participants. From a design view, this could structure what Hakken et al. (2008) call a *community of practice*, a group of people who share a concern and learn how to perform a task more effectively as they regularly interact and build design knowledge.

### Introspection for understanding

Design to prepare for discomfort and support individuals and groups to make sense of it (Schraefel et al., 2020) is in accordance with a more hypervigilant T1D runner profile (see insight 2). In this outcome, the patient’s view was considered for a better collection of self-observational and self-reflective data through analytic autoethnography, becoming a co-creator and an enhancer of data compilation that was significant for T1D self-management (when intensive PA is involved). Hence, *analytical autoethnography in design* can be recommended to help structure approaches for thinking about a particular aspect of a problem, pushing the understanding and ensuring a broader range of possibilities have been addressed. Furthermore, with the T1D runner – as well as in diabetes healthcare as Inns & Mountains have described (Inns and Mountain, 2020) *–* what is often required is working within very complex systems that need careful unpacking before we suggest improvements and change. Thus, the design management perspective aids the integration of information gathered from the running experience – and this avoids data perception as a threat.

### Learning from the experience

Chronic illnesses are associated with uncertainty boosting depression and anxiety symptoms (Hoth et al., 2015), a finding which is echoed above in Insight 3. Design management perspectives imply problem-based learning; for diabetes patient education, this provides a tool for uncertainty reduction strategies. Reducing and excising ambiguity is part of a designer’s practice. From a pragmatic point of view, design aims to solve problems (Borja de Mozota, 2003) and manage uncertainty. One of the most robust design tools for reducing uncertainty are prototypes or representations of a design idea, which help explore or demonstrate some aspect of the future artifact (Houde and Hill, 1997). The *journey map* is a mode of experience representation built through the iterative training process; it is here recommended as a strategy to reduce uncertainty during PA in T1D (see insight 3). Similarly, Chetty et al. (2019) declare the importance of building a framework for PA accomplishment in young people, including carbohydrate intake, insulin bolus, and exercise time and intensity, all of which can be prompted by *journey map design* as shown in this study to accomplish endurance PA. This design tool encourages patients to apply *problem-based learning* that (according to Funnell et al., 2007) promotes the development of relevant skills to solve self-identified problems in a simulated “real-world” environment, such as conceptual reasoning, empathy for different viewpoints, communication, collaborative working styles, and self-directed learning.

### Prototyping for empowerment

To give the patient enough confidence to solve their needs for diabetes management and improve their diabetes empowerment (see insight 4), prototyping as a design recommendation could help test and iterate self-management strategies, adjusting elements integrated in the journey map, such as insulin bolus, carbohydrate intake and glucose levels. As previously stated in Practice Design for Empowerment and Self-Management recommendations (Funnell and Anderson, 2004), prototyping is most effective once providers and their team have defined and expressed a shared vision of diabetes care and education, which can then be used to guide practice redesign (Funnell and Anderson, 2004) in order to adapt it to patient-specific needs. Echoing this idea, Kanstrup et al. (2015) previously demonstrated the impact of prototyping in relation to diabetes in order to co-create solutions and possible actions in a safe way. In addition, prototypes support participants in understanding the dynamics of the elements of the diabetes clinic models for example, a nutritional data prototype that was able to give information from service providers regarding ingredients in unlabeled food.

Ethnography to close gaps between T1D and PA: With available technology, T1D people are more involved in their self-health care and consequently demand more information, putting higher pressure on developing design strategies and on healthcare systems to successfully fill the gap between what patients expect and current healthcare practices (Frascara et al., 2018). Using patients’ complex journeys to exercise development (insight 5) as a design tool helps to bridge that gap: this places user experience and empathy at the heart of healthcare improvement projects and innovation, which can then be analyzed and measured by process mapping (Inns and Mountain, 2020), boosting self-observation and T1D self-management t.

In this regard Mamykina et al. (2006), through an ethnographic observational study, use a “culture probe” or activity framework that is useful in understanding the correlation between daily activities and blood sugar levels, suggesting the patient must become a detective of their condition, in order to “proactively engage in the analysis of their disease.” Similarly, the outcomes of the present research related to journey map fit with a design ethnography study, including both descriptive and prospective dimensions, which reflect in actions to commit in their T1D understanding for the running practice.

### Archetyping glucose profiles

Blood glucose awareness training (Cox et al., 2001; Delamater et al., 2001) is an empirically validated cognitive-behavioral therapeutic intervention that teaches patients to identify their idiosyncratic symptoms of hypo-and hyperglycemia as an adjunct to self-monitoring of blood glucose and has been shown to decrease anxiety in patients with T1D (Groot et al., 2016). Considering glycaemia as a polysemic concept for T1D runners (see insight 6), it would be useful to align glycaemia’s objective and subjective/perceptual dimensions. Thus, archetyping could be helpful both for building glycemic profiles that integrate glycaemia perception for runners and for promoting anxiety control around glucose monitoring during exercise execution. Just as patient archetypes evolve, according to O’Connor (2002) it would also be consistent for glycaemia archetypes to change according to PA type, time and intensity, as well as life stages with T1D. For example, one archetype for glycaemia could be determined by the level of interference it has with the activity, because this could force the athlete to conclude or interrupt a competition or training session.

These design recommendations are proposed for use by healthcare providers and patients looking for better diabetes management for runners following the Empowerment and Self-Management recommendations for Design Practice proposed by Funnell et al. (2007) (Table 2). All of the above can make tangible records, visualize, test and better integrate T1D data for decision making, diabetes empowerment and minimizing the barriers that the condition imposes for PA, hoping to enrich physical culture in the T1D population.

**Table 2:** Design recommendation to encourage self-management for T1D runners.

Design recommendation	Self-management strategies
Communities of practice	Involving the group of T1D runners in problem-solving, promoting opportunities for exchange experiences and co-create diabetes management solutions.
Analytical autoethnography	Auto-critic and self-reflexive analysis of their experience boost setting behavioral goals, reinforcing the T1D runner as an expert on their own support needs and making them responsible for daily self-management decisions. This requires a thorough record process of data collection.
Identity mapping	Creating opportunities for self-knowledge and diabetes acceptance, in order to facilitate social and emotional support.
Problem solving	Confirming the ability of T1D runners to identify their own problems and determine an approach to diabetes self-management that will work for them.
Prototyping	Providing information to nurture informed decision making throughout the lifetime of diabetes systematizing the trial-and-error process and boosting patients’ decision making.
Experience journey mapping	Integrating and visualizing multiple factors that affect diabetes management for a T1D runner, including clinical, psychosocial, and behavioral aspects which is a complex process.
Archetyping	Respecting the cultural background and beliefs of the T1D runners to connect with their needs.

## Data availability statement

The data that support the findings of this study are available upon request from the corresponding author DM.

## Ethics statement

The studies involving human participants were reviewed and approved by Universidad Católica de Chile Ethics Committee (ID 190725002). The participants provided their written informed consent to participate in this study.

## Author contributions

DM-B and RS: conceptualization. DM-B, KD-C, RS, and SC-C: formal analysis, writing original draft and writing review and editing. DM-B: funding acquisition, methodology, project administration, resources, and supervision. DM-B, KD-C, and SC-C: validation. DM-B and KD-C: visualization. All authors contributed to the article and approved the submitted version.

## Funding

This work was supported by Fondo Nacional para el Fomento del Deporte granted by the Chilean Ministry of Sport [grant 1900120559] and the School of Design Pontificia Universidad Católica de Chile. The authors declare that this study also received funding from Medtronic [ERP-2019-12085]. The funder was not involved in the study design, collection, analysis, interpretation of data, the writing of this article, or the decision to submit it for publication.

## Conflict of interest

The authors declare that the research was conducted in the absence of any commercial or financial relationships that could be construed as a potential conflict of interest.

## Publisher’s note

All claims expressed in this article are solely those of the authors and do not necessarily represent those of their affiliated organizations, or those of the publisher, the editors and the reviewers. Any product that may be evaluated in this article, or claim that may be made by its manufacturer, is not guaranteed or endorsed by the publisher.
