# Supplementary material for: Domesticating the condition: Design lessons gained from a marathon on how to cope with barriers imposed by type 1 diabetes
Source: Front Psychol. 2022 Nov 7;13:1013877. doi: 10.3389/fpsyg.2022.1013877 (PMC9677098; doi:10.3389/fpsyg.2022.1013877)
Supplement: Supplementary file 1 [file Data_Sheet_1.PDF]

Supplementary method

Deep interview questions

How would you describe yourself? What benefits does sport bring you? What are the main risks to your health when doing sports? What is your weekly sports routine? How is your protocol for a training/competition (before, during, and after sports, detail regarding the administration of insulin, carbohydrates, and intensities)? Who helped you put together your routine? What is the most difficult thing about exercising with DT1? Where and how is the place where you do your sports practice?; What is your diet regarding your sports practice? What is diabetes for you? Where do you look for information for sports and diabetes? Why did you decide to be an athlete? What are the main costs of sports and diabetes? Who supports your sports practice? Are there any concerns in your environment about the fact that you do high-intensity sports? Do you share your experience with people who live the same as you? Which is your best/worst sports and diabetes memory? What is your next sport´s challenge, and what do you need to achieve it?.
