# Supplementary material for: Domesticating the condition: Design lessons gained from a marathon on how to cope with barriers imposed by type 1 diabetes
Source: Front Psychol. 2022 Nov 7;13:1013877. doi: 10.3389/fpsyg.2022.1013877 (PMC9677098; doi:10.3389/fpsyg.2022.1013877)
Supplement: Supplementary file 2 [file Data_Sheet_2.PDF]

SUPPLEMENTARY FIGURES AND TABLES

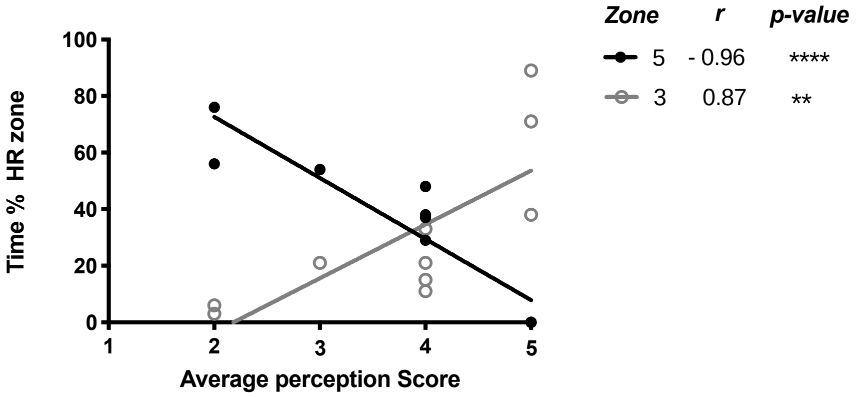

Supplementary Figure 1. Glucose management perception score correlates with the percentage of time staying in heart-rate zones three and five.
Perception score I measured in training phase 2 (during training). Spearman Correlation ****$P < 0.0001$. **$P < 0.01$.

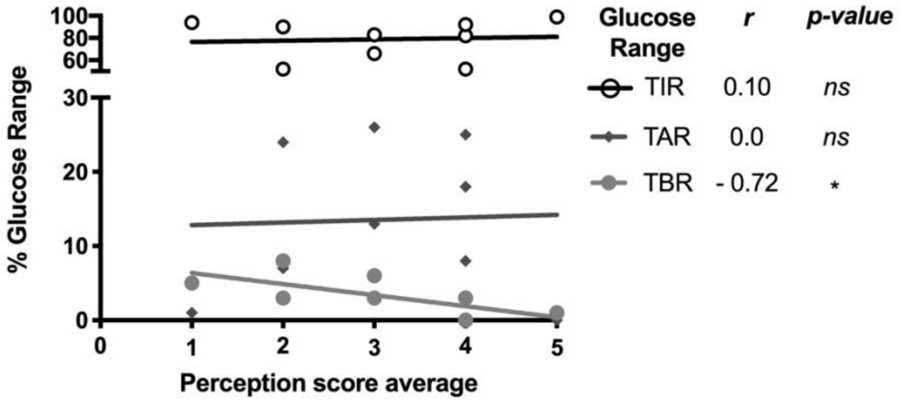

Supplementary Figure 2. Perceptions of athletes correlates inversely with the % of time spent on glycemia below range. Perception score I measured in phase 5 of training (post-training day). Spearman Correlation, *$P < 0.05$.

Supplementary Table 1. Variables considered for each training Phase

Training Phases	1- Pre training	2- During training	3- Immediately the training has finished	4- One hour post Training	5- During post-training day	6- Post-training night
Variables	✓ Carbs<br>✓ Basal Insulin<br>✓ Time<br>✓ Perception P1	✓ Time in Heart Rate Zone 5<br>✓ Time in Heart Rate Zone 4<br>✓ Time in Heart Rate Zone 3<br>✓ Time in Heart Rate Zone 2<br>✓ Time in Heart Rate Zone 1<br>✓ Pace<br>✓ Time (min)<br>✓ Kms<br>✓ Basal Insulin<br>✓ Perception P2	✓ Insulin Bolus<br>✓ Basal Insulin<br>✓ Time<br>✓ Perception P3	✓ Carbs<br>✓ Insulin Bolus<br>✓ Time<br>✓ Perception P4	✓ Time<br>✓ Perception P5	✓ Basal Insulin<br>✓ Analyse Time<br>Perception P6

Supplementary Table 2. Hypoglycemia perception score mean according to training phases

Training Phases	0- Previous night	1- Pre Training	2- During Training	3- Immediately the training has finished	4- One hour post-Training	5- During post-training day	6- Post-training night
Perception Score Mean	2.2	3.3	3.2	2.2	2	1.8	2.7
